# FDA approved drugs as potential Ebola treatments

**DOI:** 10.12688/f1000research.6164.2

**Published:** 2015-03-10

**Authors:** Sean Ekins, Megan Coffee

**Affiliations:** 1 Collaborations in Chemistry, 5616 Hilltop Needmore Road, Fuquay-Varina, NC, 27526, USA; 2 Center for Infectious Diseases and Emergency Readiness, University of California at Berkeley, 1918 University Ave, Berkeley, CA, 94704, USA

**Keywords:** FDA approval, repurposed drugs, antivirals

## Abstract

In the search for treatments for the Ebola Virus, multiple screens of FDA drugs have led to the identification of several with promising *in vitro* activity. These compounds were not originally developed as antivirals and some have been further tested in mouse *in vivo* models. We put forward the opinion that some of these drugs could be evaluated further and move into the clinic as they are already FDA approved and in many cases readily available. This may be important if there is a further outbreak in future and no other therapeutic is available.

As the Ebola outbreak continues and the costs spiral ^1^ we should perhaps be considering what alternative treatments are close to hand in Africa to complement the public health measures that have been used to date ^2^. Two independent studies funded by the US Defense Threat Reduction Agency in 2013 identified FDA approved drugs worthy of further evaluation. This work now seems prescient although it appears to have not been followed through to any public conclusion.

In one study, the antimalarials amodiaquine and chloroquine ( Figure 1) were found to be active using *in vitro* cell culture assays and an *in vivo* mouse model ^3^. Both drugs are cheap, generally safe, and likely readily accessible in Africa. These compounds have also shown relatively broad activity against other viruses *in vitro* and *in vivo* in animal models (Dengue, Coronavirus OC43, SARS etc.) ^4– 7^. A second study suggested selective estrogen receptor modulators (SERM) clomiphene and toremifene ( Figure 1) as inhibitors of Ebola virus ^8^. The latter compounds are likely more accessible in the west and indicates that other FDA or EMEA approved drugs may be worth testing including those with hormonal effects that are SERMs. More recent work from 2014 in Europe identified a further 3 FDA drugs, amiodarone, dronedarone and verapamil ( Figure 1) that inhibit filovirus entry at plasma levels attainable in humans ^9^. The mechanism of action for most of these drugs is unknown although, using computational methods we have recently shown that the antimalarials and SERMs may share some pharmacophore features which may be important to infer a potential common target or targets ^10^. To our knowledge likely well over 100 small drug-like molecules have now been identified with activity against the Ebola virus including over 50 FDA drugs derived from a reporter assay at NCATS ^11– 15^.

**Figure 1.**
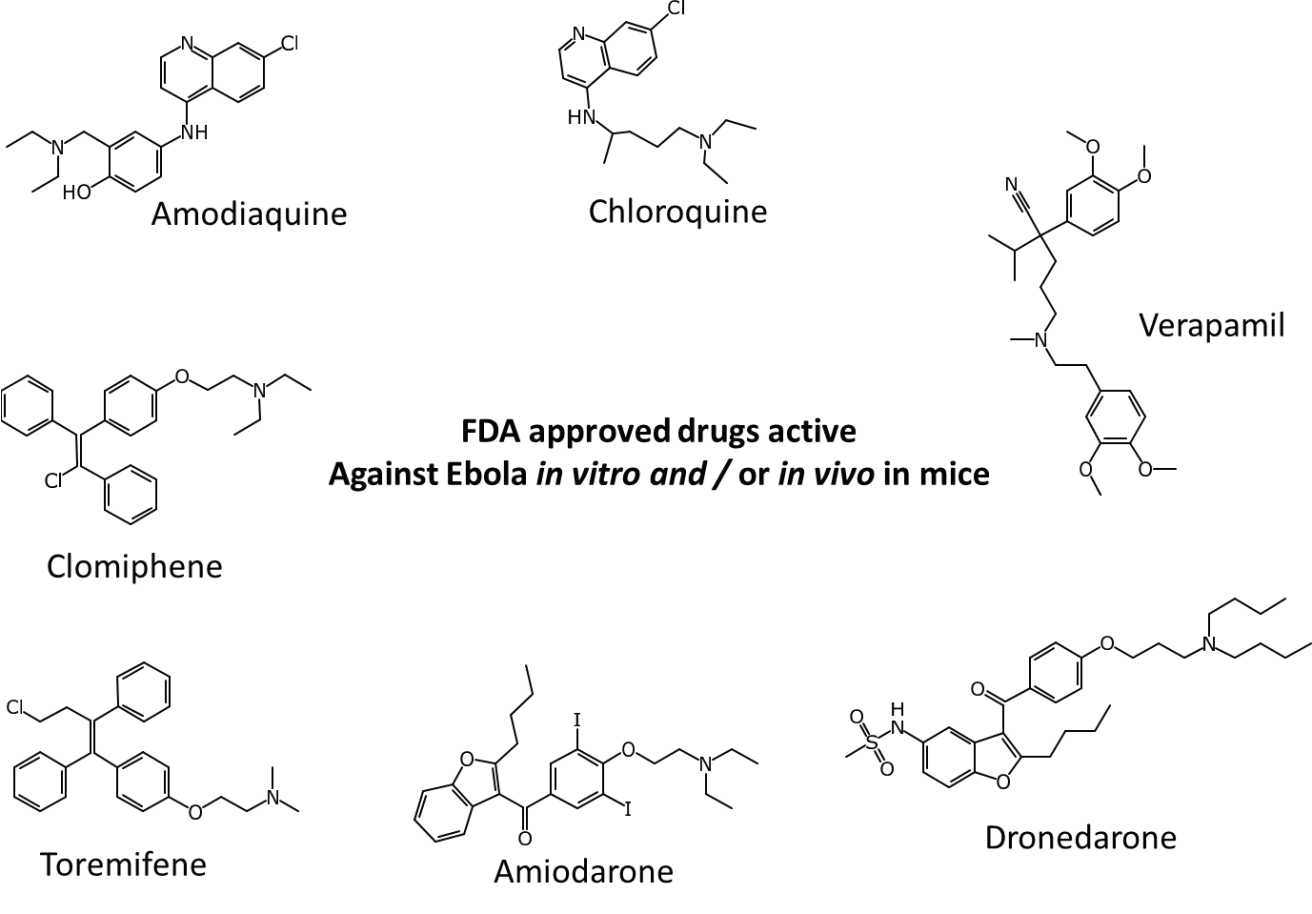
FDA approved drugs of most interest for repurposing as potential Ebola virus treatments.

As we await the development of a vaccine or biologic could we consider assessing the efficacy of the antimalarials or the other ‘FDA approved drugs’, as either treatments or prophylactics to prevent the Ebola virus from spreading further? While there can be no guarantee they will work (perhaps requiring adjusted dosage) they may be a last resort. It is possible there are other “non-antivirals” that are widely used in Africa that may also be effective against Ebola. Another example of where ‘non-antiviral’ FDA approved drugs have been found to have ‘anti-viral activity’ is for Hepatitis Virus B and D where the sodium taurocholate co-transporting polypeptide (NTCP) was identified as a receptor ^16^ and screening produced drugs such as azelastine, pioglitazone, glyburide, irbesartan and ezetimibe that inhibited the transporter and may provide potential treatments ^17, 18^. Of these compounds, azelastine has been shown to possess *in vitro* activity against Hepatitis Virus B to date ^18^.

The aforementioned screens of ‘FDA approved drugs’ ^3, 8, 9^ for Ebola virus activity, were far from comprehensive, covering only some of the known approved drugs currently in use. In an age where drug repurposing is in vogue ^19– 23^ and it can be facilitated by computational methods ^24– 26^, it would seem a valuable resource for finding compounds active against the Ebola virus. For example, the recent pharmacophores developed for Ebola ^10^ and virtual screens ^11^ could be used to computationally search larger datasets of FDA approved drugs and prioritize additional compounds for testing *in vitro*. Even using the known actives ( Figure 1) to perform simple similarity searches in a set of over 1300 Approved Drugs in a mobile app ( http://molmatinf.com/approveddrugs.html) could prioritize further compounds for testing ( Figure S1– Figure S7). For example molecules with structural similarity to chloroquine ( Figure S1) not only includes known actives like amodiaquine and hydroxychloroquine ^3^ but also suggests the antimalarials primaquine, halofantrine and the antihistamine chlorpheniramine. Molecules with similarity to amodiaquine include the kinase inhibitors neratinib and gefitinib while other kinase inhibitors have been suggested as having activity against Ebola virus ^15^, these may not be readily accessible in Africa. Other compounds retrieved by similarity include the antimicrobial pentamidine ( Figure S3, Figure S4, Figure S7), the antiemetic trimethobenzamide ( Figure S3– Figure S7) and the antihistamine doxylamine ( Figure S5). Certainly more sophisticated and exhaustive searches than this could be tried. Deciding which molecules to use or test should also involve the physician’s perspective ^27^. Alternative treatments may also be found by studying those close to patients who may not have contracted the disease and are taking a drug for another chronic disease. Whether we can find a treatment for Ebola by serendipity is questionable but some of the published studies with known drugs might point us in the right direction of where to look. The opportunity to put already available drugs like those already identified ^3, 8, 9, 11– 14^ back on the table may be a useful tool for frontline doctors to have and is worthy of more urgent discussion and research.

## Figures and Tables

**Supplemental Figure 1.**
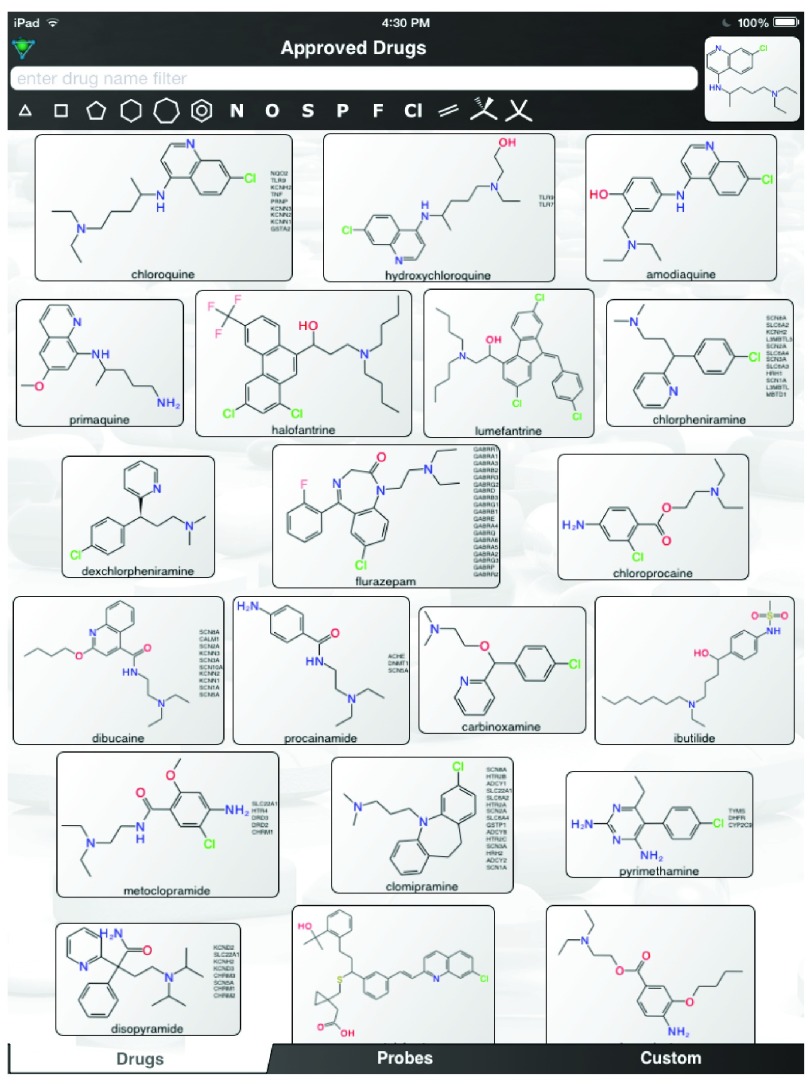
Chloroquine similarity in Approved Drugs mobile app http://molmatinf.com/approveddrugs.html.

**Supplemental Figure 2.**
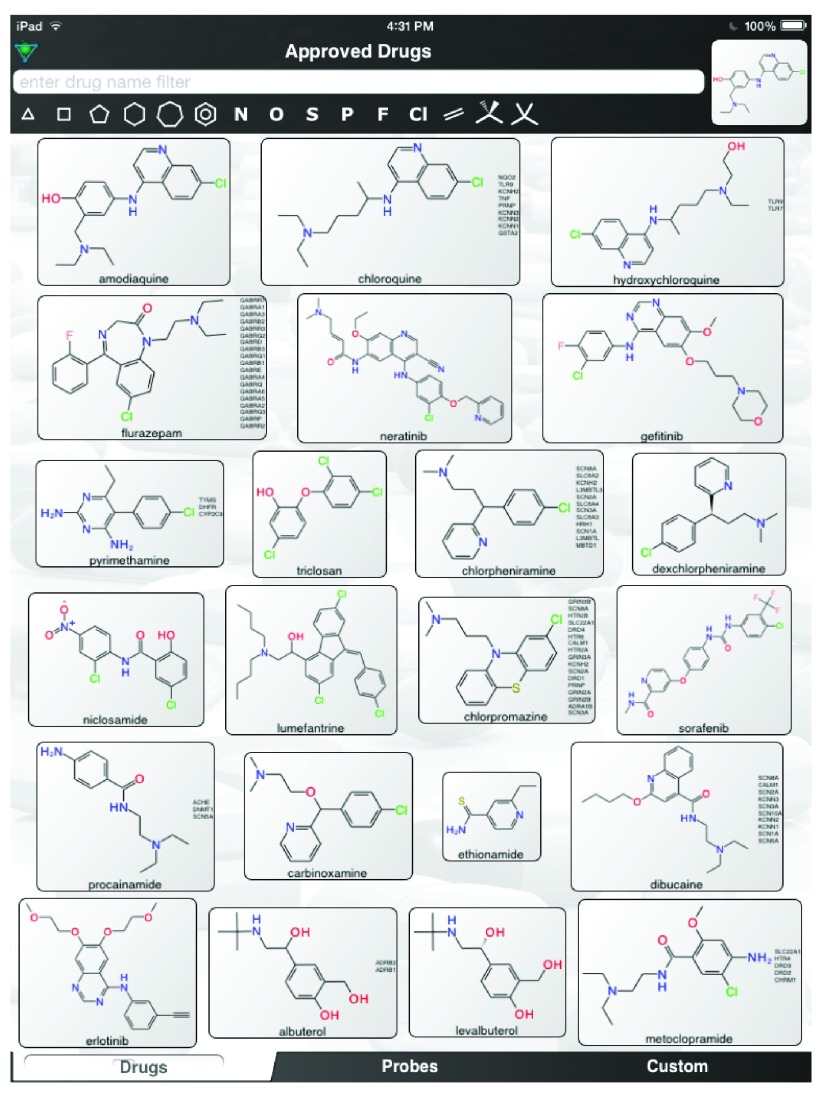
Amodiaquine similarity in Approved drugs mobile app http://molmatinf.com/approveddrugs.html.

**Supplemental Figure 3.**
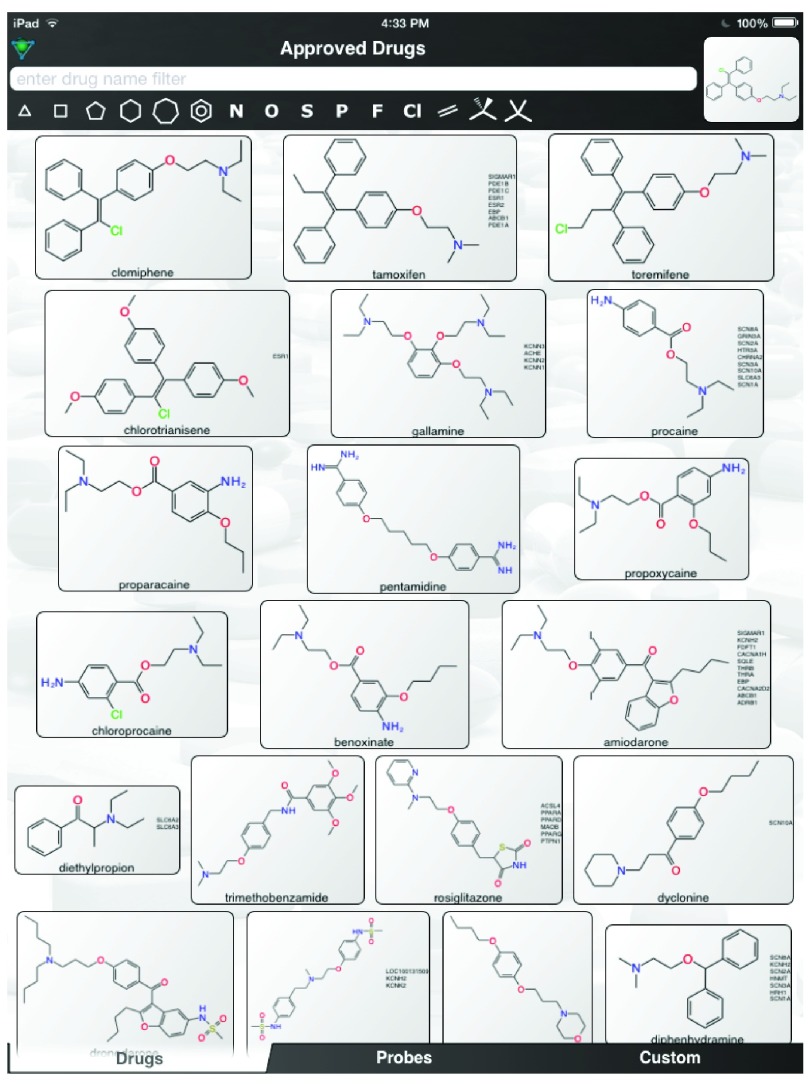
Clomiphene similarity in Approved drugs mobile app http://molmatinf.com/approveddrugs.html.

**Supplemental Figure 4.**
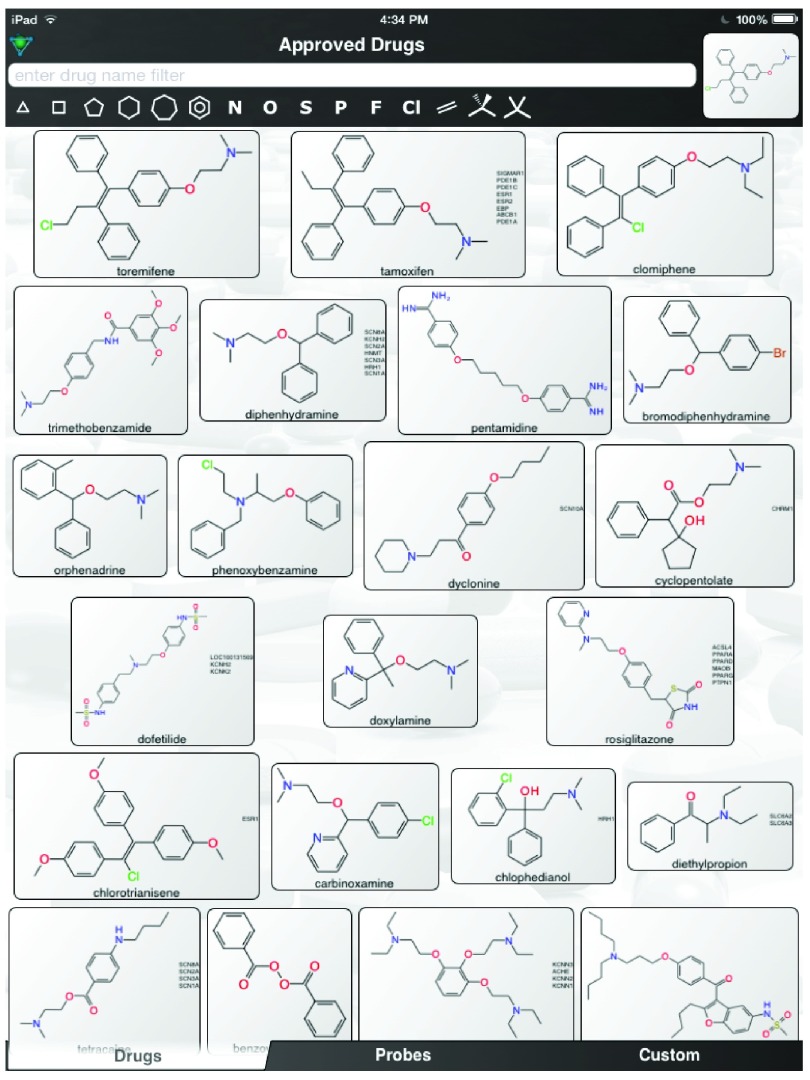
Toremifene similarity in Approved drugs mobile app http://molmatinf.com/approveddrugs.html.

**Supplemental Figure 5.**
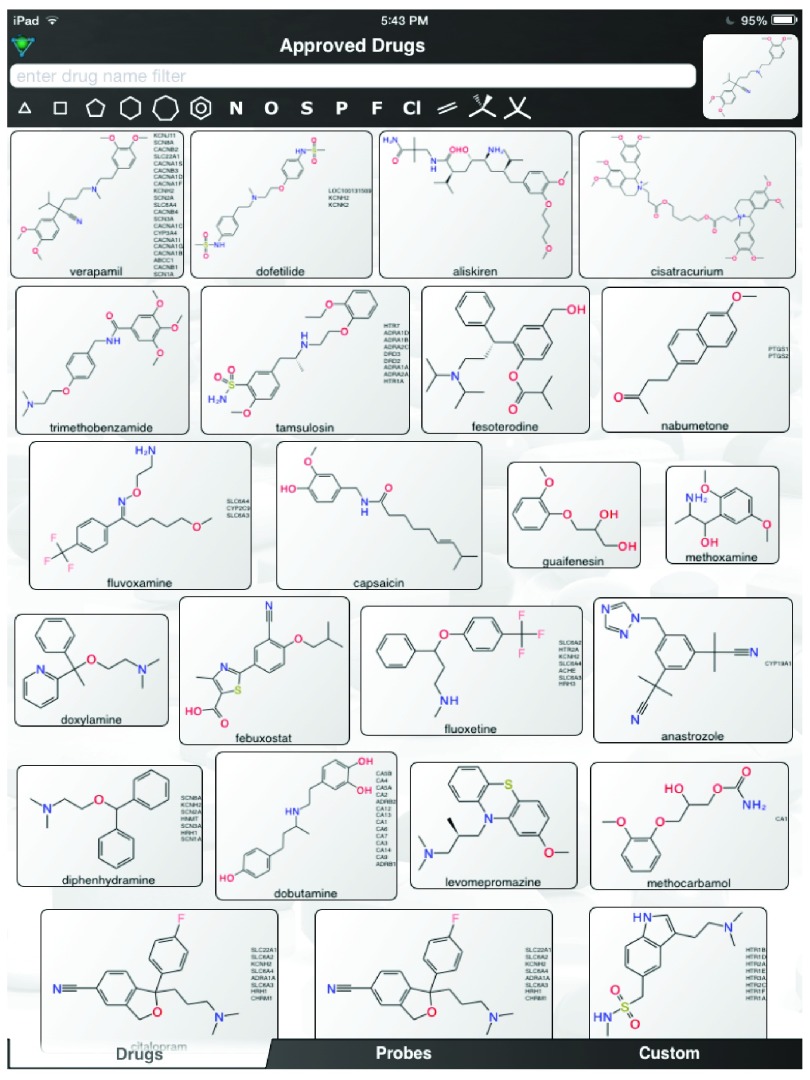
Verapamil similarity in Approved drugs mobile app http://molmatinf.com/approveddrugs.html.

**Supplemental Figure 6.**
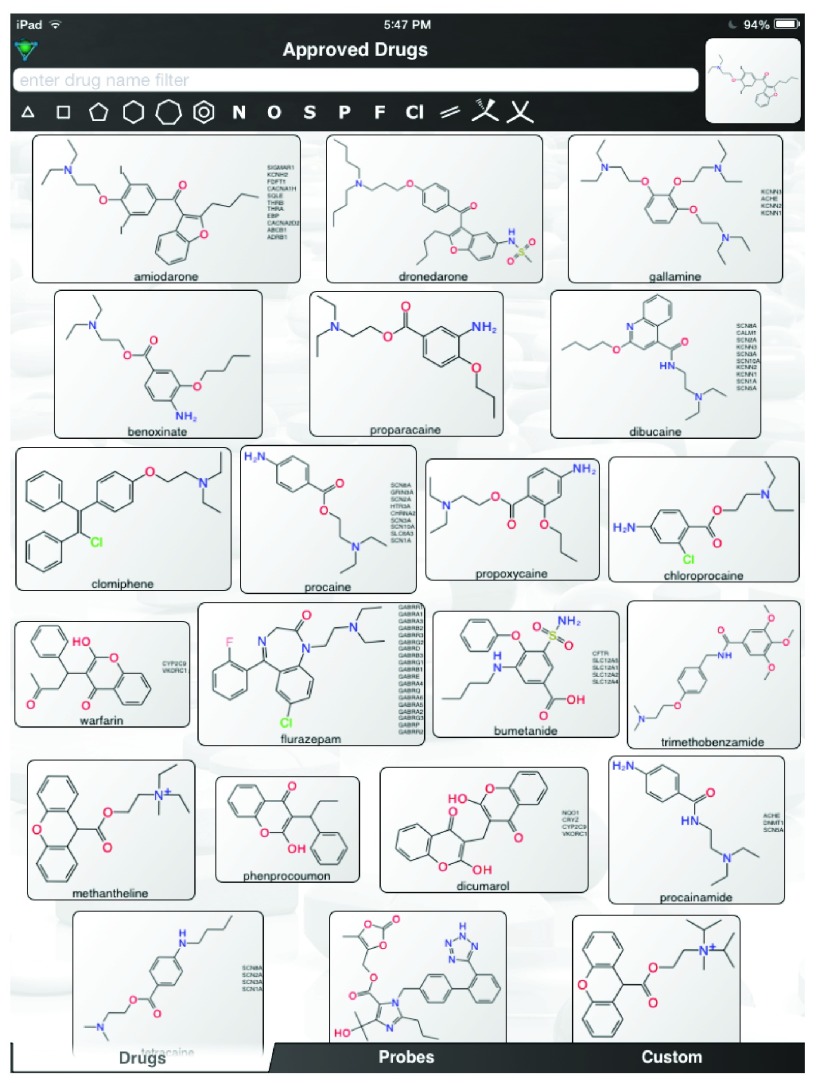
Amiodarone similarity in Approved drugs mobile app http://molmatinf.com/approveddrugs.html.

**Supplemental Figure 7.**
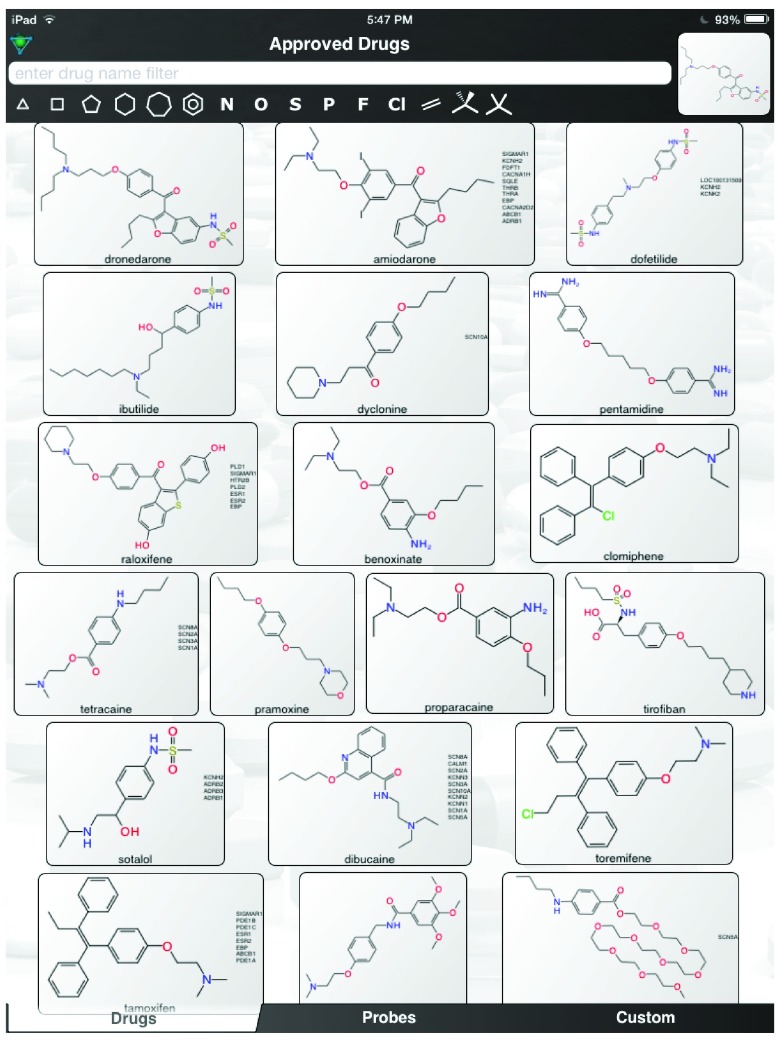
Dronedarone similarity in Approved drugs mobile app http://molmatinf.com/approveddrugs.html.
