# Supplementary material for: FDA approved drugs as potential Ebola treatments
Source: F1000Res. 2015 Mar 10;4:48. Originally published 2015 Feb 19. [Version 2] doi: 10.12688/f1000research.6164.2 (PMC4358410; doi:10.12688/f1000research.6164.2)
Supplement: Peer review history

# Peer review: Referee response for version 2

**DOI:** 10.5256/f1000research.6664.r7908

**Published:** 2015-03-11

**Authors:** James Popp

**Affiliations:** 1 Stratoxon LLC, Lancaster, PA, USA

Additions included in the revised version have improved the submission.

I have read this submission. I believe that I have an appropriate level of expertise to confirm that it is of an acceptable scientific standard.

# Peer review: Referee response for version 1

**DOI:** 10.5256/f1000research.6608.r7744

**Published:** 2015-02-27

**Authors:** James Popp

**Affiliations:** 1 Stratoxon LLC, Lancaster, PA, USA

This Opinion Article provides an interesting and potentially important view that currently approved drugs may have activity against the Ebola virus that would allow rapid entry into clinical use due to the previous approved status.  While this concept is consistent with previous scientific discussions of the potential for “repurposing” of drugs, the focus on the Ebola virus is very germane to the immediate medical crisis and the need for effective therapies related to Ebola infections. The presented opinion provides a high level overview of previously published data identifying agents with potential efficacy and the opinion appropriately expands the concept to using computational approaches to identify other drugs with potential activity. The concept of studying Ebola virus exposed individuals who did not contract the disease as an approach to identify drugs that may have a beneficial effect is very good although fraught with difficulties when such studies may be attempted under “field” conditions. This point should be expanded.

The authors are encouraged to give additional thought and provide additional opinion regarding the approach(s) that can or should be taken beyond the identification of drugs that may have potential efficacy in an Ebola outbreak. Since the opinion recommends additional screening of drugs for potential efficacy, how will (should) decisions be made to select specific agents for further evaluation or clinical use? What criteria should be deemed essential to make decisions in a selection process? These are critical issues since limited resources (and they will always be limited) will require decisions as to which molecules will be prioritized in the selection for the next level of evaluation or use.

I have read this submission. I believe that I have an appropriate level of expertise to confirm that it is of an acceptable scientific standard, however I have significant reservations, as outlined above.

# Response

**Published:** 2015-03-02

**Authors:** Sean Ekins

**Affiliations:** Collaborations in Chemistry, USA

Thank you for your review. In the latest version, the compounds from figure 1 with known ebola virus activity in vitro and in vivo, were used as a starting point for similarity searching >1300 FDA approved drugs in a mobile app (the same type of approach could likely be taken with other software). This would suggest several FDA approved molecules that could be readily tested and may be accessible in Africa (e.g. additional antimalarials and antimicrobials etc). While its unclear if these have been tested to date this type of approach could be taken on a larger scale. It may also point to the importance of a tertiary amine in these compounds for their mechanism.

In our most recent opinion http://f1000research.com/articles/4-58/v1 we discuss using the physician's perspective to group treatments which addresses the reviewers question of what criteria may be essential.

# Peer review: Referee response for version 1

**DOI:** 10.5256/f1000research.6608.r7764

**Published:** 2015-02-23

**Authors:** Raymond Lin

**Affiliations:** 1 Communicable Diseases Division, National Public Health Laboratory, Ministry of Health, Singapore, Singapore

This is a different take on the approach to therapeutics for Ebola. The idea of using non-antivirals as potential therapeutics has been broached before, and it is natural to extend that proposition to Ebola. The authors provide a good summary of some candidate agents and the laboratory evidence to suggest it might be worth a try.  Although the mechanistic explanation is not available, one mechanism which is common to some of them is by their effect on cell membrane transport through pores. The use of this class of drugs would also largely overcome some ethical issues which pertain to experimental drugs. Of course, in practice, the conduct of clinical trials would be more challenging than might appear. The finding of appropriate cases and controls, and the fact that mortality seems also largely determined by early access to supportive measures like re-hydration- these will complicate the ability to detect an outcome difference. On the subject of re-repurposing of drugs, we note also that some non-Ebola antivirals might be re-purposed for Ebola e.g. favipiravir, which has been approved for influenza in some countries.

I have read this submission. I believe that I have an appropriate level of expertise to confirm that it is of an acceptable scientific standard.

# Response

**Published:** 2015-03-02

**Authors:** Sean Ekins

**Affiliations:** Collaborations in Chemistry, USA

Thank you for your review and comments. We also mention favipiravir and other non-Ebola antivirals in our recent review http://f1000research.com/articles/4-38/v1.
